# Regression analyses of questionnaires in bedside teaching

**DOI:** 10.1186/s12909-020-02295-y

**Published:** 2020-10-16

**Authors:** Wolf Ramackers, Julia Victoria Stupak, Indra Louisa Marcheel, Annette Tuffs, Harald Schrem, Volkhard Fischer, Jan Beneke

**Affiliations:** 1 General, Visceral and Transplant Surgery, Hannover Medical School, Hannover, Germany; 2 Transplantation Centre, Management-Team, Hannover Medical School, Hannover, Germany; 3 Department of General, Visceral and Transplant Surgery, Medical University Graz, Graz, Austria; 4 Office of the Dean of Studies, Hannover Medical School, Hannover, Germany

**Keywords:** Student evaluation, Student survey, Multivariable regression, Bedside teaching, Quality management

## Abstract

**Background:**

Students’ ratings of bedside teaching courses are difficult to evaluate and to comprehend. Validated systematic analyses of influences on students’ perception and valuation of bedside teaching can serve as the basis for targeted improvements.

**Methods:**

Six hundred seventy-two observations were conducted in different surgical departments. Survey items covered the categories teacher’s performance, student’s self-perception and organizational structures. Relevant factors for the student overall rating were identified by multivariable linear regression after exclusion of variable correlations > 0.500. The main target for intervention was identified by the 15% worst overall ratings via multivariable logistic regression.

**Results:**

According to the students the success of bedside teaching depended on their active participation and the teacher’s explanations of pathophysiology. Further items are both relevant to the overall rating and a possible negative perception of the session. In comparison, negative perception of courses (worst 15%) is influenced by fewer variables than overall rating. Variables that appear in both calculations show slight differences in their weighing for their respective endpoints.

**Conclusion:**

Relevant factors for overall rating and negative perception in bedside teaching can be identified by regression analyses of survey data. Analyses provide the basis for targeted improvement.

## Background

The quality of clinical teaching is fundamental for the proficient education of future physicians. Bedside teaching, where students learn essential skills under the supervision of experienced clinicians, is a central part of clinical teaching [1–3]. Bedside teaching is a patient-centered part of medical training involving case discussions and instructions in clinical skills with real patients. Basic medical skills like history taking and physical examination are often trained in skills labs and with actor patients [4, 5]. Furthermore, bedside teaching provides additional valuable experience by learning important practical skills under professional supervision [1, 2]. Diagnostic abilities can be enhanced [6].. Recent studies have underlined the relevance of bedside teaching as teaching method despite the boom of other learning formats like e-learning [3].

As professional medical educators we strive to excel at clinical teaching. Bedside teaching strongly benefits from a good teaching climate, clear structures and adaptation to learners’ levels of skills and expertise. Clinical educators should communicate learning goals clearly and connect their teaching sessions with existing knowledge. They should observe their students carefully, supply useful feedback and encourage soft skills like students’ self-reflection and self-directed learning.

Student evaluation is a feasible way of collecting feedback [7–9] and medical teachers should utilize this strong option for continuous improvement [10, 11].

Teaching in a clinical environment is challenging. Clinical educators not only have teaching obligations, but are in charge of patient care, ward organization and personnel [7]. Still, continuous improvement in teaching quality is essential for the education of young physicians and feedback from student surveys can provide valuable information for the teacher. Therefore, a feedback tool should be easy to apply and deliver reliable data with distinct information such as most relevant factors for effective improvement.

Questionnaires are an efficient method to obtain feedback from students [12, 13]. Structured student evaluation is a feasible way of using subjective statements as valid measuring tools [12], though validity might be compromised by the teacher’s personal performance [13–16].

Given solid sample sizes, objective information can be obtained from collected opinions. Analyses and interpretation provides the basis of improvement of teaching [8, 17]. Multivariable regression analyses establish a hierarchy of the effect questionnaire items on a selected endpoint such as the overall rating or distinctly negative perception of teaching sessions [18, 19]. Identification and weighing of independent factors influencing student ratings can be derived by multivariable regression analysis and hence used as targets for focused improvement measures [18–20].

Statistical methods can identify variables which are generally relevant for the overall rating (by linear regression) and critical for distinctly negative perception (by binary logistic regression) [19, 20]. In other words, linear regression tells us what is generally important and binary logistic regression tells us what contributed to an unfavourable outcome – when choosing “worst 15%” as the outcome.

Information on the effect of each element on the overall rating provides hints for the importance of an item for the overall success: the higher parameter estimates are, the stronger is the effect on the rating of a teaching session. To focus on aspects which most urgently need improvement in a specific setting, Odds Ratios for negative perception (worst 15%) can distinguish variables strongly connected to bad ratings from those that are of general importance. Higher Odds Ratios represent a higher impact on the probability of negative perception [19].

However, assessment of survey data in bedside teaching courses is challenging in comparison to standardized education settings like seminars or lectures. The aim of this study is to utilize advanced statistical methods for a better understanding of key elements for our teaching in a specific setting.

## Methods

### Study setting

Fourth year medical students were asked to participate in a total of 14 surgery teaching sessions between April 29th 2015 and June 24th 2016 in groups of approximately 4 students.

These bedside formats of Hannover Medical School, designed to learn surgical practical skills, are taught by experienced surgical residents. Students volunteered to evaluate the courses anonymously immediately after taking part, telling their subjective perceptions as well as giving marks.

### Definition of variables and categories

Parameters were classified in three different categories for predictors (Table 1): in addition to teaching performance and organization, students rated their own contribution to the session, including their medical knowledge prior and after the lessons. Grades ranged from 1 (best) to 6 (worst) rating. Categorized predictors aimed to assess the different dimensions of bedside teaching to deliver detailed information on separate aspects and to enable targeted improvement.

**Table 1:** Statistic results of student evaluations (*n* = 672). All items were subjectively evaluated as grades from 1 to 6 with 1 being the best possible grade. Overall rating ranged from 0 to 15 points with 15 as best rating. Univariable regression was performed both linear with the endpoint overall rating and also binary logistic with the endpoint negative perception. For linear univariable regression, parameter estimates ± their standard deviation are displayed (maximum likelihood estimates). For binary univariable regression, the odds ratio with its 95% confidence interval is shown (Wald)

		Descriptive	Univariable regression	
Category	Variable	Median (IQR) or n (%)	vs. overall rating (linear)	vs. negative perception (binary)
Teacher	Feedback	1 (1–2)	−1.236 ± 0.071; *p* < 0.001	1.613 (1.353–1.923; *p* < .001)
	Pathophysiology	1 (1–1)	−1.037 ± 0.079; *p* < 0.001	2.756 (2.036–3.729; *p* < 0.001)
	Presentation of content	1 (1–1)	−1.341 ± 0.158; *p* < 0.001	3.987 (2.424–6.556; *p* < 0.001)
	Learning goals met	1 (1–2)	−0.959 ± 0.060; *p* < 0.001	2.798 (2.143–3.653; *p* < 0.001)
	Supervision	1 (1–3)	−0.322 ± 0.039; *p* < 0.001	1.332 (1.130–1.569; *p* = 0.001)
	Friendliness	1 (1–1)	−1.145 ± 0.224; *p* < 0.001	2.165 (1.07–4.378; *p* = 0.050)
	Punctual beginning	1 (1–1)	−0.573 ± 0.080; *p* < 0.001	1.579 (1.226–2.033; *p* = 0.001)
Student	Active participation	1 (1–1)	−1.380 ± 0.158; *p* < 0.001	2.474 (1.523–4.018; *p* < 0.001)
	Increase of interest	2 (1–3)	−0.599 ± 0.044; *p* < 0.001	1.814 (1.498–2.197; *p* < 0.001)
Structure	Structure	1 (1–2)	−1.236 ± 0.071; *p* < 0.001	3.596 (2.595–4.983; *p* < 0.001)
	Learning goals defined	1 (1–2)	−0.678 ± 0.046; *p* < 0.001	2.09 (1.73–2.525; *p* < 0.001)
	Ward personnel	1 (1–2)	−0.691 ± 0.057; *p* < 0.001	1.976 (1.624–2.403; *p* < 0.001)
Endpoints	Overall rating	13 (12–14)	–	–
	Negative perception	94 (13.99%)	–	–

### Handling of missing data

Returned surveys without an overall grade were excluded from analyses as they lack the defined endpoint. Missing values for predictors were imputed with the worst possible grade 6, as it either indicates a non-rateable performance or absence of the referring item.

### Study endpoints

Two different endpoints were used for respective calculations. Both a linear and a binary endpoint were calculated. The subjective overall grading of the teaching sessions by the students ranged from 0 to 15 points with 15 points representing the best possible overall grade. It was used as the continuous endpoint variable as it resembles the German school grading system. In addition, the worst 15% were transformed to the binary endpoint of ‘negative perception’. In our cohort, the most negative 15% ratings equal 11 or fewer points. This was selected as a cut-off to identify items that most urgently require improvement steps.

### Statistical testing

For descriptive statistics, all data sets were used. Regression analyses were only performed for full data sets without any missing data. Descriptive statistics cover median and interquartile range as well as total range for continuous variables, count and percentage of total for binary variables. For group comparisons, *p*-values in continuous variables were either computed by Wilcoxon-rank-sum test because of non-normally distributed data. Normal distribution was assessed by Kolmogorov-Smirnov-test and Shapiro-Wilk-test. Binary data was compared between groups by Pearson’s Chi^2^-test. Significance of results was assumed at p-values lower than 0.05.

Regression analyses included linear regression when applied to the linear endpoint of total rating or logistic regression when applied to the binary endpoint of negative perception. Univariable regression analyses were performed for all variables to identify their potential influence on the endpoints. Significance levels of 0.250 eligible for the inclusion to multivariable regression analyses which were performed by the backwards likelihood elimination method. Before inclusion, variables were tested collinearity by Pearson’s Correlation (Supplementary Table 2). When collinearity with r > 0.500 was found, only the variables with the lower *p*-value was selected for the next step. If multiple *p*-values were lower than 0.001, the variable with the higher parameter estimate or Odds Ratio was chosen. Multivariable regressions were performed for each three categories of variables separately. Odds Ratios are presented with 95% confidence interval, parameter estimates with their standard deviation.

All calculations were computed by SAS Enterprise Guide 7.1 (SAS Institute, Cary, NC, USA).

## Results

**Fig. 1:**
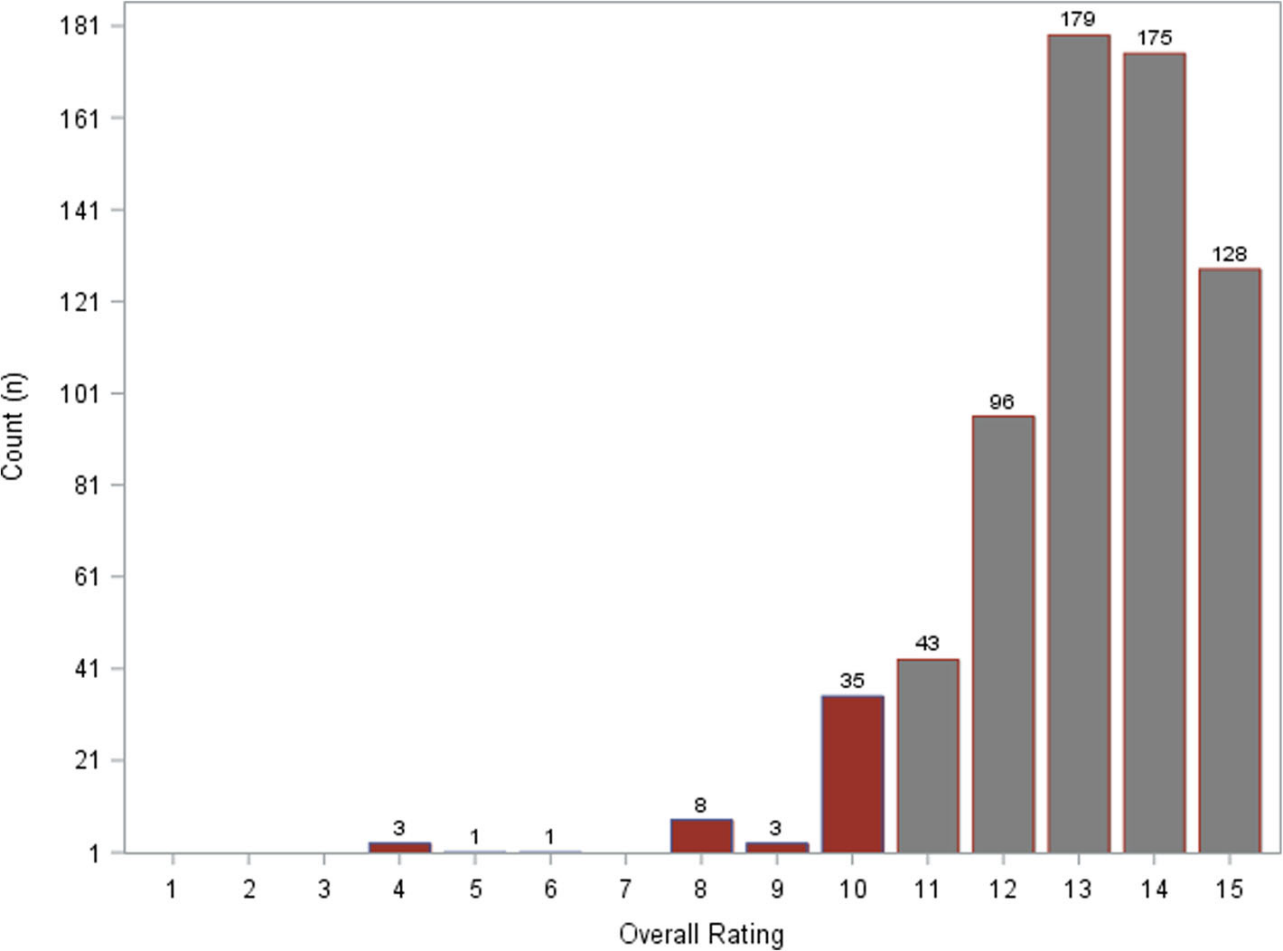
Distribution of overall ratings of students’ ratings of evaluated medical teaching sessions from 0 to 15 points with 15 points representing the best possible evaluation. Bars are labeled with the total count of ratings for each grade. The grey bars represent the best 75% (> = 11 points) and the red bars the worst 15% (< 11%)

A total of 672 questionnaires on the quality of surgical bedside teaching sessions were analysed. Students’ overall ratings ranged from 0 to 15 points (median 13, IQR 12–14) and were not normally distributed (Fig. 1). Defined as the 15% worst ratings, the binary study endpoint ‘negative perception’ equalling 11 or fewer points overall rating was observed in 13.99% (94 of 672).

Descriptive statistics are shown in Table 1. Some of the variables (teacher: explanation of pathophysiology, presentation of content, friendliness, punctuality of teacher: active participation) showed an exceptionally narrow distribution with a median of 1 and IQR of 1–1. A comparison between the two subgroups of students with a negative perception of teaching sessions (overall rating < 11 points) returned all single questionnaire items significantly worse rated in the subgroup for negatively perceived teaching sessions (Table 2).

**Table 2:** Comparison between 94 students with negatively perceived teaching sessions (< 11 points) and 578 students with overall rating above the cut-off. As results for median with interquartile range (IQR) did not explain the significant differences (as calculated by Wilcoxon-Rank-Sum-Test) intuitively, the values for mean with standard deviation were added

Category	Variable	Median ± IQR		Mean ± SD		*p* value
		No negative perception (*n* = 578)	Negative perception (*n* = 94)	No negative perception (*n* = 578)	Negative perception (*n* = 94)	
Teacher	Feedback	1 (1–2)	3 (2–4)	1.724 ± 1.298	3.079 ± 1.699	<.001
	Pathophysiology	1 (1–1)	2 (1–3)	1.279 ± 0.595	2.229 ± 1.418	<.001
	Presentation of content	1 (1–1)	1 (1–2)	1.105 ± 0.351	1.471 ± 0.612	<.001
	Learning goals defined	1 (1–2)	3 (2–5)	1.715 ± 1.109	3.457 ± 1.735	<.001
	Supervision	1 (1–3)	2 (1–5)	2.076 ± 1.608	3.051 ± 1.973	<.001
	Friendliness	1 (1–1)	1 (1–1)	1.063 ± 0.274	1.157 ± 0.367	0.005
	Punctual beginning	1 (1–1)	1 (1–2)	1.274 ± 0.747	1.706 ± 1.006	<.001
Student	Active participation	1 (1–1)	1 (1–2)	1.089 ± 0.347	1.333 ± 0.683	<.001
	Increase of interest	2 (1–3)	3 (2–4)	1.99 ± 1.234	3.286 ± 1.607	<.001
Structure	Structure	1 (1–1)	2 (1–3)	1.306 ± 0.587	2.49 ± 1.475	<.001
	Learning goals met	1 (1–2)	3 (2–4)	1.451 ± 0.766	2.909 ± 1.597	<.001
	Ward personnel	1 (1–2)	2 (1–3)	1.478 ± 0.932	2.725 ± 1.601	<.001
Endpoints	Overall rating	13 (13–14)	10 (8–10)	13.401 ± 1.174	9.098 ± 1.688	<.001

Both linear and binary logistic univariable regression analyses were performed. Targeted endpoints are the overall rating of the session (0–15 points) and the binary negative perception as 15% worst ratings (Table 1). Notably, all variables that returned *p*-values below 0.05 in univariable binary logistic regression also returned significant p-values in univariable linear regression. Most results had a very slim chance of resulting from random effects (*p* < 0.001). However, teachers’ friendliness returned a comparably weaker effect (*p* = 0.050).

Results of the multivariable regression analyses are shown in Table 3. There were more potentially relevant factors for the overall rating than for the 15% worst ratings. For the outcome ‘negative perception’, considerably fewer variables remained significant in the final multivariable regression results compared to the final multivariable linear regression. Still, all independently significant variables from binary logistic regression (endpoint “negative perception”) were likewise significant in linear regression (endpoint “overall rating”).

**Table 3:** Multivariable regression for both the linear endpoint overall rating and binary endpoint negative perception. Both binary logistic and linear regression analyses were performed within each category. Backwards likelihood elimination method was deployed for both binary logistic and linear regression in each of the three categories. For linear univariable regression, parameter estimates ± their standard deviation are displayed (maximum likelihood estimates). For binary univariable regression, the odds ratio with its 95% confidence interval is shown (Wald). The intercept for multivariable linear regression was 15.745 ± 0.198 for teacher’s performance (*p* < 0.001), 15.292 ± 0.176 for student’s self-perception (*p* < 0.001) and 14.771 ± 0.114 for structural aspects (*p* < 0.001)

Group	Variable	Multivariable regression			
		vs. overall rating (linear)		vs. negative perception (binary logistic)	
		Estimate ± SD	*p*-value	Odds Ratio(95% CI)	*p*-value
Teacher	Pathophysiology	−0.767 ± 0.082	<.001	2.526 (1.800–3.544)	<.001
	Presentation of content	−0.640 ± 0.155	<.001	–	n.s.
	Supervision	−0.203 ± 0.036	<.001	1.254 (1.040–1.512)	0.018
	Punctual beginning	−0.324 ± 0.079	<.001	–	n.s.
Student	Active participation	−0.988 ± 0.146	<.001	1.804 (1–057-3.080)	0.031
	Increase of interest	−0.530 ± 0.044	<.001	1.736 (1.425–2.116)	<.001
Structure	Learning goals defined	−0.535 ± 0.050	<.001	1.818 (1.473–2.243)	<.001
	Ward personnel	−0.440 ± 0.059	<.001	1.667 (1.313–2.117)	<.001

Questionnaires were tested for internal consistency by Cronbach’s alpha. All items returned values > .700, indicating a robust consistency (Supplementary Table 1). In addition, Pearson-Correlation between questionnaire items was performed to identify collinearity and potential redundancy. Solely in the category teacher, a correlation coefficient of 0.692 between the variables ‘feedback’ and ‘supervision’ was observed.

## Discussion

Analyses of survey data are prone to methodological challenges. Ordinal data can be non-normally distributed due to floor or ceiling effects. In addition, some variables may show very narrowly distributed data, impacting their effects in correlation analyses. As questionnaires such as the SF-36 and their modified version are extensively validated and well developed, each survey item is likely to be generally important for teaching sessions. General meaningfulness is a valuable basis of information, yet we investigated which items would have the highest impact in a given setting. This study already showed a high level of very positive general ratings. However, in order to achieve further improvement, we analysed the most critical issues.

In this scenario, the binary endpoint “negative perception” was carefully defined by the 15% most negative overall ratings. This decision was supported both by high overall ratings in average and rather narrow distributions of the questionnaire items. Therefore, we explored which issues contributed most to low overall ratings by narrowing this endpoint down to the worst 15%.

Regression analyses helped to understand what the most important issues in the current setting were. Stepwise, the statistical methods added value to our understanding of results from the student survey. First, Cronbach’s Alpha (Supplementary Table 1) confirmed that the survey items were valid in the given setting. Second, Pearson’s correlation matrix (Supplementary Table 2) shows interconnections between items but does not suffice to conclude causation or prioritization. Third, descriptive statistics (Table 1) returned an overview and hints that overall results were comparably positive. Adding the binary endpoint negatively perceived teaching sessions would eventually help to identify most critical issues. As step 4 univariable regression analyses towards “negative perception” (Table 1) reveals all items are significantly relevant. However, this does not help to prioritize those items. In the fifth step, comparisons between negative rating and non-negative ratings (Table 2) showed that some items were significantly less favourably rated in the subgroup with negative perception. Again, this would not help to choose which of those may be most critical ones to improve on.

Finally, multivariable regression analyses returned a weighed and prioritized quantification for each questionnaire item’s influence on a negative overall rating, leading to conclusive action steps.

In bedside teaching sessions students are – often for the first time – asked to apply their accumulated theoretical knowledge to clinical practice. This first step towards medical practice is a crucial experience and an elemental milestone [21], joining the equally relevant areas of theoretical and applied knowledge. Therefore, evaluation of bedside teaching covers a greater variety of aspects than seminars or lectures. As part of clinical routine, it involves staff members and patients, presenting an additional challenge to the teacher [1, 7]. Evaluation by students, as described, can help to identify weak points in teaching quality. Likewise, evaluations itself can enhance, partly emotional reflections of students, and presents itself as important part of their learning processes and experience. Main background factors are teaching environments, the patient, the teachers themselves and the students, thus these factors are discussed first [22].

### Teacher

Teachers’ ratings not only refer to their didactic skills and medical competence, but also to their appearances and interactions with the participants [14, 23]. In this study, discussion of disease pathophysiology and provided supervision appear to be the most crucial factors for both overall rating and the risk of negative perception.

Discussing disease pathophysiology is the most efficient way of retrieving existing knowledge and providing a connection to clinical skills. This connection is of upmost importance for learning effects [24–26]. Supervision can be described as a conceptual framework including different aspects as mentoring, facilitating learning, fostering self-reflection and development of professional skills [27]. In bedside teaching, experienced clinical teachers monitor students taking patients’ histories and conducting clinical examinations. They provide feedback, answer students’ questions, give advice and ensure that learning goals are achieved. Importantly, clinical educators serve as a role model determining future patient encounters [28–30].

Although being an important aspect of supervision, the survey item ‘feedback’ alone was not significant in our analysis, indicating that the entire process of supervision is more complex. Therefore, evaluation of supervision and feedback as separate factors does not appear feasible.

### Student

Besides the major impact of the teacher and the teaching and its circumstances, students’ perceptions of the teaching and its content as well as their interests and skills are also significantly contributing to teaching outcomes [31]. In linear regression, independent highly significant factors overall rating in the ‘student’ category were the student participation and the interest in surgery.

The questionnaire item “interest in surgery” is a surrogate parameter for motivation. Without motivation, self-directed learner activities are much less efficient as they lack intrinsic motivation [17]. With increasing interest in surgery, induced by the teaching session, students become intrinsically motivated to learn more about surgery. Active participation in bedside teaching is crucial for acquiring practical skills in self determined learner activities like bedside teaching [31]. The transfer of theoretical knowledge to clinical routines can only be performed by the future physicians themselves. Voluntary practice with intermittent feedback from experts is crucial for the education of future doctors [31, 32].

Interestingly, the self-perception of medical students concurred with this presumption. In comparison to all other variables, active participation has most influence on the overall rating in this survey’s results.

### Structure

A proper learning environment has a positive impact on motivation and the learning activities [33]. The preparation of the ward for the teaching session helps to create a positive learning environment. Acceptance and support by staff members despite possible interference of teaching sessions with clinical routines proves dedication to medical teaching and provides a safe environment for learning. Furthermore, clear definition of learning goals supports successful and self-directed learner activities [25, 31]. Setting distinct targets for the lesson motivates students to aim at learning goals. Promoting learner activities is a widely accepted aspect of good teaching [31].

### Patient

Core elements of bedside teaching are patients and their relatives. Most patients experience sessions of bedside teaching positively as they recognize both the increased supervision and benefit from enhanced understanding of their own medical condition [34, 35]. Comprehensive language with fewer medical terms is especially beneficial for patients with lower degrees of education [34]. Regarding this, reception does not differ between patients and their relatives [36, 37]. Furthermore, both are influenced by previous experiences and contacts with medical staff. Those experiences and possible concerns have to be addressed by teachers before the session [38].

Interaction between patients and students has critical impact on the success of bedside teaching, therefore selection of apt patients is essential [38]. Although we did not address patient selection in our survey explicitly, active participation serves as a proxy for successful interaction between students and patients. In future versions of our survey, both patients and their relatives might be included. Ideally, their perspective should be surveyed and linked to the results of student evaluations.

### General discussion

In contrast to other items, friendliness of the teacher as well as the structure of the teaching session appeared not to be relevant. In bedside teaching sessions supervision seemed to be more important than structure. In contrast, theoretical teaching sessions presenting new information appear to profit from clear structure.

However, preparation of the learning environment, communication of learning goals and the connection between the new medical content and prior knowledge are aspects of good and structured clinical teaching sessions [7, 25, 28, 39]. These different aspects are ideally present in bedside teaching sessions and are apparently recognized by the students. Punctual beginning and presentation of content by the teacher contribute to the positive perception of a teaching session and had an influence on the overall rating but are no significant factors for negative evaluation.

While all variables mentioned above proved to have an independent significant influence on overall rating and/or negative perception, they can be weighed against each other by regarding their parameter estimates or odds ratio. Interestingly, the highest influence on overall rating is students’ active participation – which also is the main target of a successful bedside teaching [40]. Although the supervision of the teacher is significantly appreciated, its influence as measured by estimate or odds ratio is comparably lower than students’ participation. Notably, it does not provide the highest risk for negative perception in comparison to the other items.

While no correlation between active participation and increase of interest in surgery was found in this study, students striving for a surgical career might have been more actively participating in bedside teaching. Ensuring this active participation is a major task for medical educators [7, 9]. Therefore, this survey item should add to the definition of successful teaching, poor evaluation results in this item would indicate a need for intensified training of supervision for the medical educator. Teachers are obliged to specifically engage students with poor active participation by professional techniques as this is critical to successful learning of students.

Still, application of pathophysiological theory presented by the teacher is greatly appreciated as second highest influence in overall rating and as highest odds ratio for negative perception.

Ideally, evaluations and their analyses return concise information for future improvement of respective teaching sessions. Following our analysis methods, relevant factors can be derived from typical survey data. Results of binary logistic regression and linear regression analyses were compared to identify which aspects were relevant for a positive perception of the sessions and which turned out critically bad. All evaluated points were significant in the linear regression analysis, indicating that the overall rating of the teaching session is related to all of these points. These data alone do not offer a basis for improvement measures. In contrast, the best leverages to target the 15% worst ratings were the following: pathophysiology, supervision, active student participation, increase of interest, definition of learning goals and preparation of the ward personnel. Presentation of contents and punctual beginning were, in contrast to results in linear regression, no leverages for improvement.

Although the results of the AUROC-analyses support that most of the relevant factors were identified, it remains possible that not all relevant factors were recognised by this study. Correlation and interactions are not necessarily equal to causation. During the statistical analyses, the stepwise selection of relevant factors may have been very close, leaving a survey item out of the final results, despite its potentially comparable importance. Therefore, it needs to be emphasized that the presented methods present a manageable and time-efficient technique to support a focus for improvement. All results returned by regression analyses need to be reviewed carefully, especially in the case of narrow distribution of survey items.

For a successful bedside teaching course, clear communication of learning goals and a well-prepared learning environment are also necessary prerequisites [24, 25, 28, 31, 33, 41]. Students in the bedside teaching benefit from supervision by a clinical teacher involving feedback, answering questions, mentoring, supporting self-reflection and development of needed skills [41]. The teacher can additionally support learning by connecting the new information presented in the course to the known pathophysiology [24]. Active participation of the students is essential for a bedside teaching course that inspires the students, and motivates them to further learning [31, 40–42].

Regression statistics utilized in our study deliver systematic analyses of evaluation results and offer systematic feedback for the medical educators to improve on their teaching. This feedback should be timely after the teaching session. Regarding the quantity of data in evaluations, automated mechanisms would be needed to scale such calculations to a whole faculty. Computational algorithms and their implementation in machine learning logic would be a feasible approach. Such algorithms and artificial intelligences are already used in pathology and diagnostic processes [43–45]. In medical education, it would be especially valuable to add the process of analyses itself to the final results, including the intermediate calculations. Selection and automatization of a thorough analyses process supports the focus on interpretation of results and on enhancement of understanding our students.

In addition to a widespread application, these methods also allow a more individualized approach that identifies strengths and weaknesses of single educators. Longitudinal observations track and support the development of teachers. When the process of survey analyses is automatized by routine algorithms, they can focus on their role of facilitators, mentors and motivators.

However, such algorithms need careful definitions by collaborating statisticians and medical educators. This study adds a novel application of regression methods to analyses of student evaluations as an exemplary and necessary step. Further methods should likewise be evaluated, such as prognostic modelling or decision forests.

### Limitations and challenges

Our study is limited by the focus on student evaluation, which excludes the perspective of patients and teachers. Although the count of observations supports an objective result, the perception of students remains a limited dimension with some associated limitations [14–16]. A cross check with other perspectives has not been performed.

Calculating a binary endpoint of 15% worst ratings from overall ratings was specifically chosen to find weak points in a generally positive setting. For different settings or questions, either the transition to a binary endpoint, the cut-off (15%) or both might need to be adjusted. In general, variables with narrow distribution for their values are at higher risk to return non-significant or even misleading results. Such variables need to be reviewed carefully, specifically by comparing regression analyses with descriptive statistics and – most importantly – checking practical plausibility by communicating with the teachers.

As regards students’ active participation, no correlation with other questionnaire items was found by Pearson’s correlation. However, it cannot be safely excluded that the individual interest and motivation of students remains a confounder for the rating of teaching sessions.

Design of questionnaires and conducting a survey influence the results [46, 47]. Results from our study are specific for the deployed questionnaire, rendering the possibility that other surveys may return differing results. As evaluations are often challenged by response rates for sufficient representativeness, thus we performed a paper-based evaluation. This approach is time-consuming and requires comparably high resources in personnel.

## Conclusion

Motivation is a key to a proficient learning experience. Motivation in teaching sessions benefits from connecting to students. Data of this survey can help with to disclose students’ reasoning from multiple perspectives – both a general overview and a distinct focus on major issues with the teaching sessions. Application of regression analyses with customized endpoints according to these perspectives adds a novel way to access this question by objective results. Presented methods that can serve as a key to unlock deeper understanding of students’ perception of teaching session and hence strengthen efforts to improve of learning experiences.

## Supplementary information

**Additional file 1.**

**Additional file 2.**

**Additional file 3.**

## Data Availability

The datasets used and analysed during the current study are available in the figshare repository: Ramackers, Wolf (2019): Bedside Teaching Evaluation Data.xlsx. Figshare. Dataset 10.6084/m9.figshare.9853124
