# Supplementary material for: Regression analyses of questionnaires in bedside teaching
Source: BMC Med Educ. 2020 Oct 16;20:371. doi: 10.1186/s12909-020-02295-y (PMC7574454; doi:10.1186/s12909-020-02295-y)
Supplement: Supplementary file 1 — Additional file 1. [file 12909_2020_2295_MOESM1_ESM.docx]

## Supplementary Table 1

| Category | Variable | Cronbachs Alpha-Coefficient (standardized) |
| --- | --- | --- |
| Teacher | Feedback | 0.714 |
|  | Pathophysiology | 0.719 |
|  | Presentation of content | 0.731 |
|  | Learning goals defined | 0.713 |
|  | Supervision | 0.719 |
|  | Friendliness | 0.739 |
|  | Punctuality | 0.740' |
| Student | Active participation | 0.730 |
|  | Increase of interest | 0.720 |
| Structure | Structure | 0.710 |
|  | Learning goals met | 0.700 |
|  | Ward personnel | 0.722 |
| Endpoints | Overall rating | 0.818 |

Supplementary Table 1: Cronbach’s standardized Alpha-Coefficient for all variables.
