# Supplementary material for: Regression analyses of questionnaires in bedside teaching
Source: BMC Med Educ. 2020 Oct 16;20:371. doi: 10.1186/s12909-020-02295-y (PMC7574454; doi:10.1186/s12909-020-02295-y)
Supplement: Supplementary file 2 — Additional file 2. [file 12909_2020_2295_MOESM2_ESM.docx]

## Supplementary Table 2

| **Pearson's Correlation Coefficients** | | | | | | | | | | | | |
| --- | --- | --- | --- | --- | --- | --- | --- | --- | --- | --- | --- | --- |
|  | Feedback | Patho-physiology | Presen-tation of content | Learning goals met | Supervision | Friendliness | Punctual beginning | Active participation | Increase of interest | Structure | Learning goals def. | Ward personnel |
| Feedback | 1 | 0.239 | 0.229 | 0.457 | 0.692 | 0.160 | 0.249 | 0.222 | 0.277 | 0.334 | 0.322 | 0.306 |
| Pathophysiology | 0.239 | 1 | 0.295 | 0.533 | 0.182 | 0.208 | 0.159 | 0.341 | 0.395 | 0.382 | 0.437 | 0.351 |
| Presentation of content | 0.229 | 0.295 | 1 | 0.354 | 0.189 | 0.295 | 0.121 | 0.292 | 0.207 | 0.311 | 0.289 | 0.239 |
| Learning goals met | 0.457 | 0.533 | 0.354 | 1 | 0.357 | 0.262 | 0.298 | 0.328 | 0.395 | 0.579 | 0.585 | 0.379 |
| Supervision | 0.692 | 0.182 | 0.189 | 0.357 | 1 | 0.131 | 0.202 | 0.186 | 0.346 | 0.296 | 0.290 | 0.242 |
| Friendliness | 0.160 | 0.208 | 0.295 | 0.262 | 0.131 | 1 | 0.085 | 0.408 | 0.208 | 0.203 | 0.150 | 0.125 |
| Punctual beginning | 0.249 | 0.159 | 0.121 | 0.298 | 0.202 | 0.085 | 1 | 0.088 | 0.137 | 0.333 | 0.193 | 0.327 |
| Active participation | 0.222 | 0.341 | 0.292 | 0.328 | 0.186 | 0.408 | 0.088 | 1 | 0.228 | 0.269 | 0.241 | 0.172 |
| Increase of interest | 0.277 | 0.395 | 0.207 | 0.395 | 0.346 | 0.208 | 0.137 | 0.228 | 1 | 0.378 | 0.389 | 0.322 |
| Structure | 0.334 | 0.382 | 0.311 | 0.579 | 0.296 | 0.203 | 0.333 | 0.269 | 0.378 | 1 | 0.538 | 0.546 |
| Learning goals def. | 0.322 | 0.437 | 0.289 | 0.585 | 0.290 | 0.150 | 0.193 | 0.241 | 0.389 | 0.538 | 1 | 0.419 |
| Ward personnel | 0.306 | 0.351 | 0.239 | 0.379 | 0.242 | 0.125 | 0.327 | 0.172 | 0.322 | 0.546 | 0.419 | 1 |

Supplementary Table 2: Correlation matrix for Pearson’s Correlation Coefficient to test for interactions between variables. Between the variables ‘Feedback’ and ‘Supervision’, a correlation of 0.692 was found.
