# Supplementary material for: Regression analyses of questionnaires in bedside teaching
Source: BMC Med Educ. 2020 Oct 16;20:371. doi: 10.1186/s12909-020-02295-y (PMC7574454; doi:10.1186/s12909-020-02295-y)
Supplement: Supplementary file 3 — Additional file 3. [file 12909_2020_2295_MOESM3_ESM.pdf]

Evaluation of Bedside Teaching in surgery

This questionnaire was developed by the department of General, Visceral and Transplant Surgery in cooperation with the Deans Office, Division of Evaluation from Hannover Medical School

Name of the Teacher: Date of the Bedside Teaching:

**The Teacher was**	Total agree					Total disagree
… punctual.	☐	☐	☐	☐	☐	☐
… presented the content certain.	☐	☐	☐	☐	☐	☐
… supervised the clinical examination well.	☐	☐	☐	☐	☐	☐
… gave me useful feedback.	☐	☐	☐	☐	☐	☐
… was friendly.	☐	☐	☐	☐	☐	☐
… verified if learning goals were met.	☐	☐	☐	☐	☐	☐
… explained pathophysiology	☐	☐	☐	☐	☐	☐

**Skills:**	Very good	good	satisfactory	fair	poor	insufficient
How were your skills before the bedside teaching?	☐	☐	☐	☐	☐	☐
How were your skills after the bedside teaching?	☐	☐	☐	☐	☐	☐

Please turn around!

General questions:

	Total agree					Total disagree
Clear learning goals were defined.	☐	☐	☐	☐	☐	☐
I could participate actively during the Bedside Teaching	☐	☐	☐	☐	☐	☐
The ward personnel were helpful.	☐	☐	☐	☐	☐	☐
The Bedside Teaching had a clear structure.	☐	☐	☐	☐	☐	☐
The Bedside Teaching improved my interest in surgery.	☐	☐	☐	☐	☐	☐

This should be changed:

____

____

____

Overall Rating of the Bedside Teaching

How do I rate the Bedside Teaching overall? (0 points = fail <> 15 points = excellent)

**0**	**1**	**2**	**3**	**4**	**5**	**6**	**7**	**8**	**9**	**10**	**11**	**12**	**13**	**14**	**15**
☐	☐	☐	☐	☐	☐	☐	☐	☐	☐	☐	☐	☐	☐	☐	☐

Additional remarks:

____

____

____

Inital letter surname mother	Month of birth mother	Inital letter surname father	Month of birth father	Your gender	Your year of birth	Year when you started your study
☐	☐☐	☐	☐☐	☐	☐☐	☐☐
